# Potential Probiotic Yeasts Sourced from Natural Environmental and Spontaneous Processed Foods

**DOI:** 10.3390/foods9030287

**Published:** 2020-03-04

**Authors:** Alice Agarbati, Laura Canonico, Enrica Marini, Emanuele Zannini, Maurizio Ciani, Francesca Comitini

**Affiliations:** 1 Department of Life and Environmental Sciences, Polytechnic University of Marche, 60131 Ancona, Italy; a.agarbati@pm.univpm.it (A.A.); l.canonico@univpm.it (L.C.); enrica.marini@staff.univpm.it (E.M.); m.ciani@univpm.it (M.C.); 2 School of Food and Nutritional Sciences, University College Cork, Cork T12 K8AF Ireland; e.zannini@ucc.ie

**Keywords:** probiotic yeasts, indigenous yeasts, natural environment, spontaneous processed foods

## Abstract

In the last decades, there has been a growing interest from consumers in their food choices. Organic, natural, less processed, functional, and pre-probiotic products were preferred. Although, *Saccharomyces cerevisiae* var. *boulardii* is the most well-characterized probiotic yeast available on the market, improvement in probiotic function using other yeast species is an attractive future direction. In the present study, un-anthropized natural environments and spontaneous processed foods were exploited for wild yeast isolation with the goal of amplifying the knowledge of probiotic aptitudes of different yeast species. For this purpose, 179 yeast species were isolated, identified as belonging to twelve different genera, and characterized for the most important probiotic features. Findings showed interesting probiotic characteristics for some yeast strains belonging to *Lachancea thermotolerans*, *Metschnikowia ziziphicola*, *Saccharomyces cerevisiae,* and *Torulaspora delbrueckii* species, although these probiotic aptitudes were strictly strain-dependent. These yeast strains could be proposed for different probiotic applications, such as a valid alternative to, or in combination with, the probiotic yeast *S. cerevisiae* var. *boulardii*.

## 1. Introduction

Historically, nature represented the primary source of most pro-technological microorganisms, used today for industrial applications in pharmaceuticals, foods, beverages, and the agrochemical industry. Although the search for new strains with unexplored properties continues, today’s investigations in this field are going through a revolution [1]. The vast number and variety of bioactive molecules isolated from microbial natural products has greatly contributed to the improvement of human well-being and health during the past century. Studies of fungal communities from specific environments have indicated non-anthropized natural environments or food processing matrices as a natural source of microbial isolation [2].

Spontaneous fermentation, and other traditional methods of processing and preserving food and beverages using unselected microorganisms, is frequently practiced around the world [3]. Until the 1960s, this practice was necessary due to the lack of knowledge and the scarce availability of well-characterized commercial starter strains to be applied in industrial processes. Today, after some forty years, all industrial fermentation in the food field is managed using starters, which have reached their maximum application. Today, a new trend can be observed: a return to the past, with increasing production of “artisanal” foods, and the promotion of high quality ingredients (some local or sustainable) intended as natural products. This spontaneous processed food represents a good ecological source for the isolation of wild microorganisms, such as fermenting yeasts, to be further investigated for their potential beneficial and/or bioactive role [4,5]. Nowadays, foods and nutrients with physiological health benefits for humans and animals are collectively defined as “health/functional food” (HFF) [6]. There has been a focus on the marketing of foods supplemented with live microorganisms, in adequate amounts, able to provide beneficial effects for the hosts, such as regulation of intestinal microbial balance, immune modulation, and reduction of inflammatory bowel diseases [7,8,9,10]. These microorganisms are defined as probiotics by the Food and Agriculture Organization of the World Health Organization (WHO) [11]. For a long time, most of the researchers focused on the use of bacteria as probiotic microorganisms, while yeast remained poorly investigated in this field [12,13]. In the first half of the 20th century, Henri Boulard isolated a yeast species from lychee fruit named *Saccharomyces boulardii* [14], actually classified as *Saccharomyces cerevisiae* var. *boulardii* [15,16], and, subsequently, studied its probiotic properties. Currently, it represents the most common human probiotic yeast studied in detail and available on the market [13]. This yeast is recommended for the prevention and treatment of human gastrointestinal diseases, such as ADD (antibiotic associated diarrhea) and IBD (inflammatory bowel disorders), and the control of serum cholesterol, chronic diarrhea in immunodeficient patients (AIDS-related diarrhea), and acute diarrhea in adults and children. It also seemed to have positive effects in the treatment of *Clostridium difficile* and *Helicobacter pylori* infections [16,17,18,19,20,21]. The yeast represents a good alternative to probiotic bacteria because it is immune to the antibiotic effect, can avoid the antibiotic-associated human intestinal diseases [16], can reduce the use of antibiotics, and, therefore, limit the development of antibiotic resistance. These reasons led researchers to focus their attention on the study of other yeast species with probiotic properties. Indeed, some strains belonging to the *Debaryomyces*, *Kluyveromyces*, *Yarrowia,* and *Torulaspora* genus were recently proposed as microorganisms with potential health benefits [22,23,24,25]. For example, *Debaryomyces hansenii*, Kluyveromyces lactic, and other technological yeasts were recently approved by the EFSA (European Food Safety Authority) and included on the list of “Qualified Presumption of Safety” (QPS) microorganisms assumed safe [26]. It was described as able to confer beneficial effects in fish when used as a nutritional supplement [27]. However, to date, only *S. boulardii* is considered a probiotic yeast [12] because other “alternative” species need more definitive in vitro characterization before their utilization in human trials and applications [25].

The aim of the present study was to exploit the great microbial biodiversity of the natural environment (un-anthropized) and artisan food matrices as a source of wild yeasts with specific properties. In this regard, about 180 yeasts were isolated, selected, and identified as belonging to the *Torulaspora*, *Debaryomyces*, *Kluyveromyces*, *Candida*, *Kazachstania*, *Metschnikowia*, *Pichia*, *Hanseniaspora*, *Saccharomyces*, *Rhodotorula*, *Brettanomyces,* and *Lachancea* genus. Then, they were characterized for the most important probiotic aptitudes, with the goal to detect new possible probiotic yeasts to be placed on the market.

## 2. Materials and Methods

### 2.1. Source and Isolation Procedure of Yeasts

The isolation campaign was carried out by exploiting the yeast biodiversity of two types of sources:

(i) Natural environments (NE), such as woods, soil, fruit plats, sandstones pits, cellar and dairy, from un-atrophied sites located in the Marche region (center of Italy);
(ii) Spontaneous processed foods (SPF), such as sourdoughs, cheeses, wines, beers and sugarcane juice, spontaneously fermented.

Relatively to the NE category, the collection of microorganisms was carried out by rubbing an area of 10 cm^2^ (or 5 cm^2^ for fruits with reduced available dimensions) using a sterile non-absorbent cotton swab, as described by Biagiotti and co-workers [28]. After sampling, each swab was immediately placed into 10 mL of a sterile physiological solution. Relative to SPF, 100 g (sourdough and cheese) or 100 mL (wine, beer and sugarcane juice) of each sample was collected using a sterile bag. All the samples were maintained at 4 °C until arrival in the laboratory for processing. NE samples were then aseptically maintained overnight on a rotary shaker at 150 rpm at 4 °C, to facilitate microbial release, while sourdough and cheese samples were subjected to stomacher homogenization (25 °C for 5 min) adding 100 mL of sterile physiological solution. Ten-fold dilutions were made for all samples and spread onto Wallerstein Laboratories (WL) agar (Oxoid, Hampshire, UK) with 0.02% biphenyl to prevent mold diffusion, Rose Bengal medium (Oxoid, Hampshire, UK) with chloramphenicol to inhibit bacteria development, and Lysine Agar medium (Oxoid, Hampshire, UK)) for non-*Saccharomyces* yeasts selection. The plates were incubated at 25 °C for 5 days. Representative yeast colonies were selected on the basis of their micro- and macro-morphological differences, from the highest diluted plates of each matrix and the numbers of isolations made in relationship to the relative abundance of each plate. Pure isolates were maintained at 4 °C on YPD medium (yeast extract 1%, peptone 2%, dextrose 2%, and agar 2%) for subsequent analyses, and in YPD broth supplemented with 80% (*w*/*v*) glycerol for long-term storage at −80 °C.

### 2.2. DNA Extraction

About 180 pure yeast cultures were selected and used for the yeast’s DNA extraction, according to the method reported by Stringini et al. [29]. First, the isolates were pre-cultured on YPD agar for 3 days at 25 °C. Then, the cells were transferred to screwcap tubes containing glass beads and reaction buffer (Trizma 0.1 M, pH 8.0, EDTA (Ethane diyldinitrilo tetraacetic acid) 50 mM, SDS (Sodium dodecyl sulfate 1%). The tubes were vortexed, boiled for 10 min, and placed on ice to allow cell wall disruption. Next, 20 μL of Tris–HCl 1 M (pH 8.0), 15 μL of EDTA 0.5 M (pH 8.0), 50 μL of SDS 10%, and 200 μL of potassium acetate 5 M were added, and the tubes were incubated on ice for 30 min. After centrifugation, the supernatant containing the DNA was transferred to a new tube containing ice-cold isopropanol, incubated on ice for 5 min, centrifuged, and the pellet resuspended in ice-cold ethanol 70%. After centrifugation, the DNA was resuspended in a Tris-EDTA buffer and left at 45 °C for 15 min. The DNA obtained was stored at −20 °C until processing.

### 2.3. Yeast Species Identification

The ITS1-5.8S rRNA-ITS2 region was amplified by PCR (Polymerase Chain Reaction) using primer pair ITS1 (5′-TCCGTAGGTGAACCTCGCG-3′) and ITS4 (5′-TCCTCCGCTTTATTGATATGC-3′), as described by White and co-workers [30]. The amplification was performed in a reaction mix containing 0.5 µM of each primer, 10 µM of each dNTP, 1.5 mM of MgCl, 1 vol. of PCR buffer 10×, 1U of DreamTaq DNA polymerase (Fermentans, Thermo Fisher Scientific Inc., Waltham, MA, USA) and 5 µL of extracted DNA solutions in a final volume of 100 µL. The PCR was performed in a Biorad Thermal Cycler (Bio Rad, Hercules, CA, USA), using an initial denaturation at 95 °C for 5 min, followed by 35 cycles of 94 °C for 1 min, annealing at 55 °C for 2 min, elongation at 72 °C for 2 min, and a final extension at 72 °C for 10 min. PCR products were separated on 1.5% agarose gel stained with 0.5 mg/mL of ethidium bromide in 0.5× TBE (Tris-borate-EDTA) and visualized under UV. A Generuler 100 bp DNA ladder (Fermentans, Thermo Fisher Scientific Inc., MA, USA) was used to compare the size of the bands obtained. The sequencing approach was used to obtain sequences of the representative yeasts, which were compared with those already present in the data library using the BLAST program [31] and the GenBank database (http://www.ncbi.nlm.nih.gov/BLAST) [32] for yeast species identification.

### 2.4. Probiotic Characterization

#### 2.4.1. Ability to Grow at 37 °C

The isolates were first tested for their ability to grow at internal body temperature. The strains were pre-cultured on YPD broth for 24 h at 25 °C. They were then transferred to fresh media with an inocula of 10^6^ cells/mL. Changes in optical density were monitored after 48 h of incubation at 37 °C. The commercial probiotic *S. cerevisiae* var. *boulardii* (CODEX, Zambon Italia S.r.l., Bresso, Italy) was used as the positive control strain. The trial was conducted in duplicate.

#### 2.4.2. Effect of Low pH and Bile on Yeast’s Growth and Viability

All the isolates were evaluated for their ability to grow in the presence of bile and low pH. The strains were pre-cultured on YPD broth for 24 h at 25 °C. They were then used to set up the trials following the protocol described by van der Aa Kühle et al. [33], with some modifications. Yeast Nitrogen Base (YNB, Biolife, Milan, Italy) was acidified with HCl 2 N to reach pH 2.5, and added to 0.3% (*w*/*v*) bile salts (Merck KGaA, Darmstadt, Germany). It was used as the growth medium. An inoculum of about 10^6^ cells/mL of each pre-culture was made in duplicate, and the changes in cell density after 48 and 120 h of incubation at 37 °C were monitored by counting the cells using a Thoma–Zeiss counting chamber, as suggested by Casagrande-Pierantoni et al. [34]. Viability of the cells was estimated using methylene blue staining. YNB without bile salts, not acidified, inoculated with yeasts, and incubated at 37 °C was used as the control. The probiotic *S. cerevisiae* var. *boulardii* (CODEX, Zambon Italia S.r.l., Bresso, Italy) was used as the positive control strain.

#### 2.4.3. Antimicrobial Activity

The antimicrobial activity of the about 180 strains was assessed by the double-layer agar technique, as described by Perricone and co-workers [35]. Six microbial species potentially pathogenic for humans were used as sensitive strains: *Candida albicans*, *Escherichia coli*, *Listeria monocytogenes*, *Staphylococcus aureus,* and *Salmonella enterica*. Plate Count Broth (tryptone 5.0 g/L; yeast extract 2.5 g/L; glucose 1.0 g/L) was used to allow the bacteria’s growth twice at 30 °C for 24 h, while YPD broth was used for *C. albicans* under the same conditions. The potential probiotics were pre-cultured on YPD broth for 24 h at 25 °C, and 100 µL of the pre-culture (7 log CFU/mL) were distributed onto the surface of YPD agar plates and incubated at 30 °C for 24 h. A second soft layer of nutrient agar (beef extract 3 g/L; peptone 5 g/L; agar 15 g/L) was distributed onto the surface of YPD agar, and the potential pathogen strains were streaked on the surface of the soft layer and incubated at 37 °C for 24 h. The probiotic *S. cerevisiae* var. *boulardii* was used as the positive control strain. Plates without potential probiotics were prepared as negative controls. The antimicrobial activity of the yeasts tested was evaluated as the presence of an area of inhibition of pathogen growth.

#### 2.4.4. Antioxidant Activity

The about 180 isolates were tested for their ability to scavenge the DPPH (1,1-Diphenyl-2-Picrylhydrazyl) radical following the method described by Chen et al. [36]. All the strains were pre-cultured onto YPD broth for 24 h at 25 °C and the probiotic *S. cerevisiae* var. *boulardii* was used as positive control strain. In short, 800 µL of fresh cell solution and 1 mL of DPPH solution (0.2 mM in methanol) were mixed and left at 25 °C for 30 min. The samples were centrifuged at 2000 g-force for 2 min and the scavenged DPPH was monitored by measuring the decrease in absorbance (A) at 517 nm. The trial was conducted in duplicate and the blank sample was prepared using de-ionized water. The scavenging ability of each strain was defined by solving the following equation: [1−A_517(sample)_/A_517(blank)_] × 100%.

## 3. Results

### 3.1. Isolation and Identification of the Isolates

The primary isolation campaign was carried out in natural environments (wood, soil, fruit, plants, sandstone pits, cellars, and dairy) and spontaneously processed foods (sourdoughs, cheeses, wine, beer, and sugarcane juice), and 179 yeast strains were isolated and identified through the sequencing of ITS1-5.8S and rRNA-ITS2 regions.

The 179 sequence alignments in the GenBank database revealed the presence of Brettanomyces, Candida, Debaryomyces, Hanseniaspora, Kazachstania, Kluyveromyces, Lachancea, Metschnikowia, Pichia, Rhodotorula, Rhodosporidiobolus, Saccharomyces, and Torulaspora, and about 22 other species, as reported in Table 1.

As expected, NE samples showed greater variability of genera and, specifically, a high number of different *Metschnikowia* species, such as *pulcherrima*, *ziziphicola, fructicola,* and *reukaufi*. *S. cerevisiae* were isolated in a single case (oak moss). This supported the evidence that *S. cerevisiae* is vanishingly rare on fruit, even in vineyards where fruiting plants are at very high artificial densities and in which the associated winemaking would be expected to increase the overall abundance of yeast in the location [37]. Alternatively, an abundance of *S. cerevisiae* strains was found in SPF samples where the natural matrices were fermented. In these cases, *S. cerevisiae* obviously dominated in wine samples together with *S. bayanus,* and in sourdough samples together with *Kazachstania unispora*. Cheese matrices represented the most abundant source of *Debaryomyces hansenii* [38].

### 3.2. Probiotic Aptitudes

After identifying the 179 yeast species, the evaluation of potential probiotic characteristics was performed. For this purpose, a series of tests, growth at 37 °C (Table 2), the ability to survive at low pH, high bile concentrations of the antioxidant property (Table 3), and antagonistic behavior against human pathogens (Table 4), were carried out.

#### 3.2.1. Growth at 37 °C

The 179 yeast strains isolated and identified were evaluated for their ability to grow at 37 °C. Table 2 shows that 130 out of the 179 strains were able to survive in a condition similar to that of natural internal body temperature. In particular, a greater capacity to grow at 37 °C was observed for the strains belonging to the *Brettanomyces*, *Candida*, *Debaryomyces,* and *Saccharomyces* genus. Alternatively, only one of the seven strains identified as *Kazachstania* was able to grow at this temperature, and variable results were observed within the *Torulaspora* genus.

#### 3.2.2. Antioxidant Activity, Low pH, and Bile Effects

The antioxidant capacity of the isolated strains was measured by the DPPH method in the final medium extracts. Table 3 reports the antioxidant activities of the tested samples evaluated in comparison to CODEX strain, and used as the positive control. Results showed lower values of *D. hansenii*, *B. bruxellensis*, *H. uvarum,* and *K. unispora* in comparison with the positive control. Many strains belonging to the *T. delbrueckii* species (isolated from NE and SPF matrices) revealed higher antioxidant activity, together with *L. thermotolerans*, *L. waltii*, *Candida* spp. and *M. ziziphicola* isolated from bark or bark moss, and *P. fermentans* and *K. marzianus* coming from unpasteurized malt. This result strongly supports the importance of wood as un-anthropized natural habitat to isolate bioactive new strains [39].

The 179 strains were also evaluated for their ability to survive in chemical conditions similar to the conditions found in the gastrointestinal tract (Table 3). All strains belonging to the *Brattanomyces*, *Candida*, *Debaryomyces*, *Kazachstania*, *Saccharomyces,* and *Torulaspora* genera were able to survive at pH 2.5 with 0.3% bile salts for 48 hours. However, in comparison with CODEX, used as the positive control, the screened strains showed great variability in the percent viability. Indeed, *Candida* MMF1_1128 and MMI1_1129, *Debaryomyces* BAT2_1170, BB4_1204, LAIF1_1167, MM1_1193 and MMS1_1196, and *Saccharomyces* 4PV exhibited higher persistence than CODEX (42.7%). After 120 h of incubation at the same conditions, a general trend in viability reduction was observed for all the strains tested. The only *S. cerevisiae* 4PV maintained the 90.9% viability after 120 h of exposure to these stressful conditions.

#### 3.2.3. Antimicrobial Activity of the Isolates

The antimicrobial activity of the 179 isolates was evaluated via a double-layer agar test and is reported in Table 4. The results indicated that all the strains belonging to *Kazachstania*, *Kluyveromyces*, *Lachancea*, *Saccharomyces,* and *Torulaspora* exhibited antimicrobial activity against *C. albicans*, *E. coli*, *S. aureus,* and *S. enterica* bacteria, comparable with the results shown by the positive control, CODEX. On the contrary, most strains of *Candida* and *Debaryomyces* seemed to be unable to counteract the growth of pathogens, with the exception of *Candida* 7, 28, B9, B10, and B9 that exhibited the same antimicrobial activity of the control. Similarly, few strains of *Debaryomyces* showed results comparable to the control. As expected, *L. monocytogenes* showed the greatest resistance to the antimicrobial activity of yeasts [40].

## 4. Discussion

Nowadays, there is great interest in the design of functional foods that contain probiotic microbial strains responsible for health benefits in the host. Indeed, several studies suggest the important role of probiotic microorganisms as promoters of human health because they are involved in the modulation of immune response and in the prevention of diseases such as inflammatory bowel, gastrointestinal, and atopic disorders and allergies [9,41,42]. Moreover, a microorganism defined as potentially probiotic and one that exerts beneficial effects on human health should possess the ability to tolerate acid pH and bile salts (to survive in the gastrointestinal tract), adhere to and/or persist in the mucosal and epithelial surfaces for immune-modulation, and to exercise competitiveness and antimicrobial activity against human pathogens [9,11,43]. Most of the probiotic microorganisms belong to the genera of lactic acid producing bacteria, but some yeast strains that exist in dairy and fermented products are classified as probiotics [25]. In addition, probiotic features are strain specific and accurate screening is required for selection of truly probiotic yeasts. Different from bacteria, yeasts are microorganisms much easier to handle both in the laboratory and on the industrial scale, and their management is less expensive. Moreover, traditional fermented dairy and not-dairy foods, such as fermented vegetables, craft beer, various natural cheeses and yogurts, are useful original resources for finding novel probiotics or processed foods to which probiotics could be added to give them safety and beneficial properties [44,45,46].

Generally, natural environments represent specific and peculiar ecological niches for a great number of yeasts that survive by responding to stress conditions, such as different pH values, high osmotic pressure, and salinity, to resist the action of antibiotics and to produce active compounds [47,48].

A general protocol for yeast selection was proposed by Pulvirenti and co-workers [49], and this could be applied to the selection of yeasts with functional and probiotic aptitudes, even if studies that describe the health-promoting properties of yeast remain limited [50,51]. To reinforce the possible use of yeasts as probiotics, in the present study, the main probiotic features of wild yeast strains isolated from natural environments and spontaneous processed foods were evaluated. Regarding to the ability of the yeasts to survive at close to human body temperature (37 °C), most of the yeast strains isolated possessed this ability independent of the isolation matrix. Kumura and co-workers [22], testing probiotic applications of yeasts isolated from cheese, confirmed that species belonging to *Kluyveromyces*, *Yarrowia*, *Debaryomyces*, *Saccharomyces,* and *Candida* were able to grow at 37 °C. Regarding antioxidant activity, the strains belonging to the *Kazachstania*, *Pichia*, *Saccharomyces,* and *Torulaspora* genus showed results comparable to or greater than the control, while variable activity was observed within the strains of the other genera tested. Previous work by [36] described the excellent antioxidant activity of *Pichia* strains. The ability of the yeast to survive in chemical conditions similar to the gastrointestinal tract were widely diffused in *Brattanomyces*, *Candida*, *Debaryomyces*, *Kazachstania*, *Saccharomyces,* and *Torulaspora* strains, showing results comparable both to each other and the control strain. In this regard Zivkovic et al. [52] described *T. delbrueckii* as the most resistant strain in the gastric juice simulated conditions, highlighting a poor survival rate of *S. cerevisiae*. It was interesting also to note a wide diffusion of antibacterial activity of the *Kazachstania*, *Kluyveromyces*, *Lachancea*, *Saccharomyces,* and *Torulaspora* strains. Although no adhesion tests on intestinal cell lines have been performed, this seemed not to be a prerequisite for the potential probiotic yeasts to exhibit inhibitory action against pathogenic bacteria [33].

After this preliminary screening of the 179 tested strains, 13 yeasts belonging to *L. thermotolerans* (B13 strain), *M. ziziphicola* (B27 strain), *S. cerevisiae* (10c, 8, 6, 2PV, 7 strains), and *T. delbrueckii* (1.1t2, 7.3t2, 35, c7.4, j401, tdvcsff strains) showed better results when compared with CODEX, the widely available in the pharma market for all the probiotic features assayed.

## 5. Conclusions

In conclusion, our in vitro study demonstrated that wild yeasts from natural environment and spontaneous processed foods could represent a valid source of potential probiotic yeasts. In particular, the results highlighted the probiotic aptitudes of 13 yeasts isolated in moss on oak (*L. thermotolerans*), beech tree bark (*M. ziziphicola*), wine (*S. cerevisiae*), sugar cane juice (*T. delbrueckii*), papaya leaves (*T. delbrueckii*), wineries (*T. delbrueckii*), and grapes (*T. delbrueckii*).

Based on their features, these yeasts could be proposed, for probiotic applications, as a valid alternative to the widely available probiotic yeast *S. cerevisiae* var. *boulardii*. Further investigation is needed to clearly define the yeasts, their safety, their health-promoting efficacy, and the dosage, following the WHO criteria and EFSA recommendations.

## Figures and Tables

**Table 1:** Origin, source and identification of the 179 yeast strains isolated.

Category Sampling	Source	Identification Codes	Specie
**NE**	Apples (Italy)	G3	*Metschnikowia pulcherrima*
	Apples (Italy)	G6	*Metschnikowia pulcherrima*
	Artisan dairy1 (Italy)	LAIF1_1123	*Candida zeylanoides*
	Artisan dairy1 (Italy)	LAIF1_1124	*Candida zeylanoides*
	Artisan dairy1 (Italy)	LAIF1_1125	*Candida zeylanoides*
	Artisan dairy1 (Italy)	LAIF1_1126	*Candida zeylanoides*
	Artisan dairy2 (Italy)	AN4_1127	*Candida zeylanoides*
	Artisan dairy3 (Italy)	MM1_1132	*Candida zeylanoides*
	Artisan dairy3 (Italy)	MM2_1133	*Candida zeylanoides*
	Artisan dairy3 (Italy)	MM4_1135	*Candida zeylanoides*
	Artisan dairy3 (Italy)	MM4_1136	*Candida zeylanoides*
	Artisan dairy3 (Italy)	MM2_1140	*Candida zeylanoides*
	Artisan dairy3 (Italy)	MM2_1141	*Candida zeylanoides*
	Artisan dairy4 (Italy)	BB2_1145	*Candida zeylanoides*
	Artisan dairy5 (Italy)	PC1_1159	*Debaryomyces hansenii*
	Artisan dairy5 (Italy)	PC2_1160	*Debaryomyces hansenii*
	Artisan dairy5 (Italy)	PC2_1161	*Debaryomyces hansenii*
	Artisan dairy5 (Italy)	PC3_1163	*Debaryomyces hansenii*
	Artisan dairy5 (Italy)	PC5_1164	*Debaryomyces hansenii*
	Artisan dairy5 (Italy)	PC5_1165	*Debaryomyces hansenii*
	Artisan dairy5 (Italy)	PC5_1166	*Debaryomyces hansenii*
	Artisan dairy3 (Italy)	MM2_1177	*Debaryomyces hansenii*
	Artisan dairy3 (Italy)	MM2_1178	*Debaryomyces hansenii*
	Artisan dairy3 (Italy)	MM2_1179	*Debaryomyces hansenii*
	Artisan dairy3 (Italy)	MM3_1180	*Debaryomyces hansenii*
	Artisan dairy3 (Italy)	MM3_1181	*Debaryomyces hansenii*
	Artisan dairy3 (Italy)	MM3_1182	*Debaryomyces hansenii*
	Artisan dairy3 (Italy)	MM4_1184	*Debaryomyces hansenii*
	Artisan dairy3 (Italy)	MM6_1186	*Debaryomyces hansenii*
	Artisan dairy3 (Italy)	MM6_1187	*Debaryomyces hansenii*
	Artisan dairy3 (Italy)	MM6_1188	*Debaryomyces hansenii*
	Artisan dairy3 (Italy)	MM7_1190	*Debaryomyces hansenii*
	Artisan dairy3 (Italy)	MM7_1191	*Debaryomyces hansenii*
	Artisan dairy3 (Italy)	MM1_1193	*Debaryomyces hansenii*
	Artisan dairy3 (Italy)	MM6_1194	*Debaryomyces hansenii*
	Artisan dairy2 (Italy)	AN4_1195	*Debaryomyces hansenii*
	Artisan dairy4 (Italy)	BB1_1197	*Debaryomyces hansenii*
	Artisan dairy4 (Italy)	BB2_1199	*Debaryomyces hansenii*
	Artisan dairy1 (Italy)	LAI2_1200	*Debaryomyces hansenii*
	Artisan dairy1 (Italy)	LAI3_1201	*Debaryomyces hansenii*
	Artisan dairy1 (Italy)	LAI3_1202	*Debaryomyces hansenii*
	Artisan dairy4 (Italy)	BB4_1204	*Debaryomyces hansenii*
	Beech tree bark (Italy)	B27	*Metschnikowia ziziphicola*
	Beech tree bark (Italy)	B28	*Metschnikowia aff. fructicola*
	Beech tree bark (Italy)	B33	*Metschnikowia pulcherrima*
	Caco fruit	E1	*Pichia fermentans*
	Coconut palm (Cameroon)	15.2t2	*Torulaspora delbrueckii*
	Corrosol fruit (Cameroon)	2.2t1	*Torulaspora delbrueckii*
	Corrosol fruit (Cameroon)	34	*Torulaspora delbrueckii*
	Grapes (Spain)	101	*Lachancea thermotolerans*
	Grapes (Italy)	102	*Lachancea thermotolerans*
	Grapes (Italy)	103	*Lachancea thermotolerans*
	Grapes (Italy)	104	*Lachancea thermotolerans*
	Grapes (Italy)	105	*Lachancea thermotolerans*
	Wine (Italy)	106	*Lachancea thermotolerans*
	Farnia bark (Italy)	B42	*Metschnikowia pulcherrima*
	Farnia bark (Italy)	B49	*Metschnikowia reukaufii*
	Fig fruit (Italy)	1C	*Torulaspora delbrueckii*
	Fig fruit (Italy)	1E	*Torulaspora delbrueckii*
	Grapes (Italy)	37	*Torulaspora delbrueckii*
	Grapes (Italy)	38	*Torulaspora delbrueckii*
	Grapes (Italy)	39	*Torulaspora delbrueckii*
	Grapes (Italy)	40	*Torulaspora delbrueckii*
	Grapes (Italy)	92	*Torulaspora delbrueckii*
	Grapes (Italy)	Td vcs ff	*Torulaspora delbrueckii*
	Grapes (Italy)	2A	*Torulaspora delbrueckii*
	Grapes (Italy)	3H	*Torulaspora delbrueckii*
	Grapes (Italy)	4E	*Torulaspora delbrueckii*
	Grapes (Italy)	5D	*Torulaspora delbrueckii*
	Malt (Italy)	Md	*Kluyveromyces marxianus*
	Malt (Italy)	Mf	*Kluyvermyces marxianus*
	Malt (Italy)	Mb	*Hanseniaspora uvarum*
	Malt (Italy)	Ma	*Pichia fermentans*
	Malt (Italy)	Mg	*Pichia fermentans*
	Oak moss (Italy)	B7	*Torulaspora delbrueckii*
	Oak moss (Italy)	B8	*Lachancea waltii*
	Oak moss (Italy)	B13	*Lachancea thermotolerans*
	Oak moss (Italy)	B15	*Lachancea thermotolerans*
	Oak moss (Italy)	B57	*Lachancea thermotolerans*
	Oak moss (Italy)	B9	*Candida spp.*
	Oak moss (Italy)	B10	*Candida spp.*
	Oak moss (Italy)	B29	*Candida spp.*
	Oak moss (Italy)	B6	*Saccharomyces cerevisiae*
	Papaya leaves (Cameroon)	33	*Torulaspora delbrueckii*
	Papaya leaves (Cameroon)	C 7.4	*Torulaspora delbrueckii*
	Papaya leaves (Cameroon)	12.2t2	*Torulaspora delbrueckii*
	Papaya leaves (Cameroon)	7.3t2	*Torulaspora delbrueckii*
	Papaya leaves (Cameroon)	7.3t0	*Torulaspora delbrueckii*
	Red maple leaves (Italy)	B5	*Rhodotorula mucillaginosa*
	Soil (Italy)	6809	*Torulaspora delbrueckii*
	Thistle (Italy)	B14	*Rhodosporidiobolus spp.*
	Wall of the cheese pit (Italy)	F14	*Metschnikowia spp.*
	Wall of the cheese pit (Italy)	F5	*Rhodotorula spp.*
	Winery (Italy)	94	*Torulaspora delbrueckii*
	Winery (Italy)	J401	*Torulaspora delbrueckii*
	Winery (Italy)	LV12	*Hanseniaspora spp.*
	Winery (Italy)	LV8	*Hanseniaspora osmophila*
**SPF**	Fossa cheese (Italy)	7	*Candida zeylanoides*
	Fossa cheese (Italy)	28	*Candida homilentoma*
	Fossa cheese (Italy)	18	*Pichia anomala*
	Fossa cheese (Italy)	25	*Debaryomyces hansenii*
	Fossa cheese (Italy)	46	*Saccharomyces cerevisiae*
	Gueze beer	G2	*Brettanomyces bruxellensis*
	Gueze beer	G4	*Brettanomyces bruxellensis*
	Gueze beer	G6	*Brettanomyces bruxellensis*
	Gueze beer	G8	*Brettanomyces bruxellensis*
	Pecorino cheese3 (Italy)	MMF1_1128	*Candida zeylanoides*
	Pecorino cheese3 (Italy)	MMI1_1129	*Candida zeylanoides*
	Pecorino cheese3 (Italy)	MMI2_1130	*Candida zeylanoides*
	Pecorino cheese3 (Italy)	MMF2_1137	*Candida zeylanoides*
	Pecorino cheese3 (Italy)	MMF2_1138	*Candida zeylanoides*
	Pecorino cheese3 (Italy)	MMF1_1142	*Candida zeylanoides*
	Pecorino cheese3 (Italy)	MMS1_1143	*Candida zeylanoides*
	Pecorino cheese4 (Italy)	BAT1_1144	*Candida zeylanoides*
	Pecorino cheese5 (Italy)	PCF1_1147	*Debaryomyces hansenii*
	Pecorino cheese5 (Italy)	PCF1_1148	*Debaryomyces hansenii*
	Pecorino cheese5 (Italy)	PCF1_1149	*Debaryomyces hansenii*
	Pecorino cheese5 (Italy)	PCF2_1150	*Debaryomyces hansenii*
	Pecorino cheese5 (Italy)	PCF2_1151	*Debaryomyces hansenii*
	Pecorino cheese5 (Italy)	PCS1_1152	*Debaryomyces hansenii*
	Pecorino cheese5 (Italy)	PCS1_1153	*Debaryomyces hansenii*
	Pecorino cheese5 (Italy)	PCS2_1154	*Debaryomyces hansenii*
	Pecorino cheese5 (Italy)	PCS2_1155	*Debaryomyces hansenii*
	Pecorino cheese5 (Italy)	PCI1_1156	*Debaryomyces hansenii*
	Pecorino cheese5 (Italy)	PCI1_1157	*Debaryomyces hansenii*
	Pecorino cheese5 (Italy)	PCI2_1158	*Debaryomyces hansenii*
	Pecorino cheese1 (Italy)	LAIF1_1167	*Debaryomyces hansenii*
	Pecorino cheese1 (Italy)	LAIF1_1168	*Debaryomyces hansenii*
	Pecorino cheese1 (Italy)	LAIF2_1169	*Debaryomyces hansenii*
	Pecorino cheese4 (Italy)	BAT2_1170	*Debaryomyces hansenii*
	Pecorino cheese4 (Italy)	BAT2_1171	*Debaryomyces hansenii*
	Pecorino cheese4 (Italy)	BAT2_1172	*Debaryomyces hansenii*
	Pecorino cheese4 (Italy)	BEM1_1173	*Debaryomyces hansenii*
	Pecorino cheese4 (Italy)	BEM1_1174	*Debaryomyces hansenii*
	Pecorino cheese4 (Italy)	BEM1_1175	*Debaryomyces hansenii*
	Pecorino cheese3 (Italy)	MMF1_1176	*Debaryomyces hansenii*
	Pecorino cheese4 (Italy)	BAT1_1192	*Debaryomyces hansenii*
	Pecorino cheese3 (Italy)	MMS1_1196	*Debaryomyces hansenii*
	Pecorino cheese4 (Italy)	BES1_1198	*Debaryomyces hansenii*
	Sourdough homemade1 (Italy)	m1-1	*Saccharomyces cerevisiae*
	Sourdough homemade1 (Italy)	m1-2	*Saccharomyces cerevisiae*
	Sourdough homemade1 (Italy)	m1-3	*Saccharomyces cerevisiae*
	Sourdough homemade1 (Italy)	m1-4	*Saccharomyces cerevisiae*
	Sourdough homemade1 (Italy)	m1-5	*Saccharomyces cerevisiae*
	Sourdough homemade1 (Italy)	m1-6	*Saccharomyces cerevisiae*
	Sourdough homemade1 (Italy)	m1-7	*Saccharomyces cerevisiae*
	Sourdough homemade1 (Italy)	m1-8	*Saccharomyces cerevisiae*
	Sourdough homemade1 (Italy)	m1-9	*Saccharomyces cerevisiae*
	Sourdough homemade2 (Italy)	m2-1	*Saccharomyces cerevisiae*
	Sourdough homemade2 (Italy)	m2-2	*Saccharomyces cerevisiae*
	Sourdough homemade2 (Italy)	m2-3	*Saccharomyces cerevisiae*
	Sourdough homemade3 (Italy)	m3-4	*Kazachstania unispora*
	Sourdough homemade3 (Italy)	m3-5	*Kazachstania unispora*
	Sourdough homemade3 (Italy)	m3-6	*Kazachstania unispora*
	Sourdough homemade3 (Italy)	m3-7	*Kazachstania unispora*
	Sourdough homemade3 (Italy)	m3-A3	*Kazachstania unispora*
	Sourdough homemade3 (Italy)	m3-B3	*Kazachstania unispora*
	Sourdough homemade3 (Italy)	m3-C3	*Kazachstania unispora*
	Sugar cane juice (Cameroon)	35	*Torulaspora delbrueckii*
	Sugar cane juice (Cameroon)	19.4t0	*Torulaspora delbrueckii*
	Sugar cane juice (Cameroon)	1.1t2	*Torulaspora delbrueckii*
	Sugar cane juice (Cameroon)	19.1t2	*Torulaspora delbrueckii*
	Sugar cane juice (Cameroon)	19.2t2	*Torulaspora delbrueckii*
	Sugar cane juice (Cameroon)	19.3t2	*Torulaspora delbrueckii*
	Verdicchio wine (Italy)	7V	*Saccharomyces bayanus*
	Verdicchio wine (Italy)	8C	*Saccharomyces bayanus*
	Verdicchio wine (Italy)	10C	*Saccharomyces cerevisiae*
	Verdicchio wine (Italy)	8	*Saccharomyces cerevisiae*
	Verdicchio wine (Italy)	2	*Saccharomyces cerevisiae*
	Verdicchio wine (Italy)	5V	*Saccharomyces cerevisiae*
	Verdicchio wine (Italy)	6	*Saccharomyces cerevisiae*
	Verdicchio wine (Italy)	4PV	*Saccharomyces cerevisiae*
	Verdicchio wine (Italy)	2PV	*Saccharomyces cerevisiae*
	Verdicchio wine (Italy)	5	*Saccharomyces cerevisiae*
	Verdicchio wine (Italy)	1PV	*Saccharomyces cerevisiae*
	Verdicchio wine (Italy)	7	*Saccharomyces cerevisiae*
	Verdicchio wine (Italy)	4	*Saccharomyces cerevisiae*

NE, natural environment; SPF, spontaneous processed foods.

**Table 2:** Evaluation of the ability of each isolate to grow at 37 °C. The CODEX, Zambon Italia S.r.l., Bresso, Italy, strain was used as the positive control. The “+”expresses the order of magnitude increase in optical density, the “±” indicates faint growth, and the “−“ represents no growth.

Yeast Strains	Growth at 37 °C	Yeast Strains	Growth at 37 °C
*Brettanomyces* genus		*Kluyveromyces* genus	
G2	+	101	−
G4	+	102	−
G6	+	103	−
G8	+	104	+
*Candida* genus		105	−
7	−	106	+
28	+	Md	+
AN4_1127	+	Mf	+
B9	−	*Lachancea* genus	
B10	±	B8	+
B29	−	B13	+
BAT1_1144	+	B15	−
BB2_1145	+	B57	−
LAIF1_1123	+	*Metschnikowia* genus	
LAIF1_1124	+	B27	±
LAIF1_1125	+	B28	±
LAIF1_1126	+	B33	+
MM1_1132	+	B42	+
MM2_1133	+	B49	−
MM2_1140	+	F14	+
MM2_1141	+	G3	+
MM4_1135	+	G6	−
MM4_1136	+	*Pichia* genus	
MMF1_1128	+	18	+
MMF1_1142	+	E1	+
MMF2_1137	+	Ma	+
MMF2_1138	+	Mg	+
MMI1_1129	+	*Rhodotorula* genus	
MMI2_1130	+	B5	+
MMS1_1143	+	B14	+
*Debaryomyces* genus		F5	−
25	±	*Saccharomyces* genus	
AN4_1195	+	46	+
BAT1_1192	+	B6	+
BAT2_1170	+	m1-1	+
BAT2_1171	+	m1-2	+
BAT2_1172	+	m1-3	+
BB1_1197	+	m1-4	+
BB2_1199	+	m1-5	+
BB4_1204	+	m1-6	+
BEM1_1173	+	m1-7	+
BEM1_1174	+	m1-8	+
BEM1_1175	+	m1-9	+
BES1_1198	+	m2-1	+
LAI2_1200	+	m2-2	+
LAI3_1201	+	m2-3	+
LAI3_1202	+	7V	+
LAIF1_1167	+	8C	+
LAIF1_1168	+	10C	+
LAIF2_1169	+	8	+
MM1_1193	+	2	+
MM2_1177	+	5V	+
MM2_1178	+	6	+
MM2_1179	+	4PV	+
MM3_1180	+	2PV	+
MM3_1181	+	5	+
MM3_1182	+	1PV	+
MM4_1184	+	7	+
MM6_1186	+	4	+
MM6_1187	+	CODEX	+
MM6_1188	+	*Torulaspora* genus	
MM6_1194	−	1C	−
MM7_1190	+	1E	−
MM7_1191	+	2A	−
MMF1_1176	+	3H	+
MMS1_1196	+	4E	−
PC1_1159	+	5D	−
PC2_1160	+	B7	−
PC2_1161	+	1.1t2	+
PC3_1163	+	2.2t1	+
PC5_1164	+	7.3t2	+
PC5_1165	+	7.3t0	+
PC5_1166	+	12.2t2	+
PCF1_1147	+	15.2t2	−
PCF1_1148	−	19.1t2	−
PCF1_1149	+	19.2t2	−
PCF2_1150	+	19.3t2	−
PCF2_1151	+	19.4t0	−
PCI1_1156	+	33	−
PCI1_1157	+	34	±
PCI2_1158	+	35	±
PCS1_1152	+	37	−
PCS1_1153	+	38	±
PCS2_1154	+	39	−
PCS2_1155	+	40	+
*Hanseniaspora* genus		92	−
Mb	±	94	−
LV8	−	6809	−
LV12	−	C 7.4	+
Ctr.	+	J401	−
*Kazachstania* genus		Td vcs ff	±
m3-4	−		
m3-5	−		
m3-6	−		
m3-7	−		
m3-A3	−		
m3-B3	+		
m3-C3	−		

**Table 3:** Antioxidant activity of the 179 isolates, and pH and bile effect on growth and viability. Antioxidant activity was expressed as scavenging of DPPH (%). The pH and bile effect on the yeast’s growth and viability were monitored after 48 and 120 h of incubation; cells/mL and viability % were reported. The results were compared with the commercial *S. cerevisiae* var. *boulardii* CODEX. Results are expressed as mean ± standard deviation.

Yeast Strains	Antioxidant Activity			pH and Bile Effect															
				0 h				48 h						120 h					
	(DPPH %)			Cells/mL			Viability %	Cells/mL			Viability %			Cells/mL			Viability %		
*Brettanomyces* genus																			
G2	17.14	±	1.19	6.14	±	0.03	100	6.17	±	0.13	21.8	±	6.4	6.11	±	0.12	0.0	±	0.0
G4	23.11	±	0.65	6.02	±	0.07	100	6.06	±	0.08	27.5	±	5.3	6.14	±	0.03	0.0	±	0.0
G6	20.62	±	0.66	6.06	±	0.08	100	6.05	±	0.03	22.3	±	1.8	6.07	±	0.00	0.0	±	0.0
G8	26.40	±	0.62	6.12	±	0.13	100	6.13	±	0.07	23.6	±	3.9	6.07	±	0.06	0.0	±	0.0
*Candida* genus																			
7	36.35	±	1.11	5.67	±	0.04	100	5.72	±	0.04	13.4	±	1.3	6.17	±	0.04	0.0	±	0.0
28	24.33	±	0.20	6.17	±	0.08	100	6.05	±	0.03	16.7	±	1.3	6.03	±	0.04	0.0	±	0.0
AN4_1127	12.50	±	0.54	6.36	±	0.01	100	6.50	±	0.00	65.0	±	0.4	6.32	±	0.01	7.5	±	3.3
B9	68.18	±	1.54	6.09	±	0.09	100	6.06	±	0.08	33.0	±	6.3	6.06	±	0.05	0.0	±	0.0
B10	63.24	±	0.03	6.05	±	0.10	100	6.03	±	0.00	47.1	±	0.0	5.95	±	0.06	0.0	±	0.0
B29	69.12	±	1.00	6.22	±	0.06	100	6.13	±	0.04	9.3	±	0.9	6.17	±	0.01	4.3	±	0.1
BAT1_1144	17.78	±	0.28	6.21	±	0.00	100	6.21	±	0.00	61.4	±	0.2	5.88	±	0.00	0.0	±	0.0
BB2_1145	18.44	±	0.30	6.19	±	0.01	100	6.02	±	0.03	29.3	±	1.0	5.64	±	0.00	11.0	±	2.1
LAIF1_1123	29.66	±	0.17	6.09	±	0.00	100	6.95	±	0.70	30.4	±	1.0	6.76	±	0.00	0.0	±	0.0
LAIF1_1124	13.35	±	0.37	6.17	±	0.00	100	6.38	±	0.71	34.8	±	0.8	6.71	±	0.00	9.7	±	2.0
LAIF1_1125	13.84	±	0.20	5.88	±	0.01	100	6.09	±	0.01	15.2	±	4.2	6.54	±	0.00	18.3	±	2.4
LAIF1_1126	16.14	±	0.24	5.67	±	0.04	100	6.35	±	0.04	12.2	±	6.3	6.08	±	0.00	7.0	±	2.8
MM1_1132	8.96	±	0.11	5.71	±	0.01	100	6.30	±	0.00	53.4	±	3.7	6.62	±	0.00	43.8	±	8.8
MM2_1133	18.59	±	0.59	5.91	±	0.00	100	6.31	±	0.00	51.9	±	2.7	6.51	±	0.00	41.9	±	3.3
MM2_1140	15.18	±	1.14	6.29	±	0.00	100	6.50	±	0.00	45.0	±	7.1	6.64	±	0.00	0.0	±	0.0
MM2_1141	16.66	±	0.47	5.28	±	0.00	100	6.13	±	0.00	81.8	±	0.6	6.43	±	0.02	0.0	±	0.0
MM4_1135	16.77	±	0.32	5.70	±	0.00	100	6.05	±	0.01	40.2	±	3.8	6.45	±	0.01	18.7	±	5.0
MM4_1136	18.34	±	0.48	5.79	±	0.00	100	6.36	±	0.01	32.5	±	10.6	7.17	±	0.71	5.9	±	0.8
MMF1_1128	4.14	±	0.18	5.70	±	0.00	100	6.01	±	0.01	93.5	±	9.2	6.08	±	0.01	0.0	±	0.0
MMF1_1142	16.01	±	0.91	5.50	±	0.00	100	5.70	±	0.01	60.4	±	2.9	6.23	±	0.01	0.0	±	0.0
MMF2_1137	1.45	±	0.51	5.58	±	0.00	100	5.57	±	0.00	39.3	±	15.2	6.38	±	0.00	35.4	±	2.9
MMF2_1138	1.46	±	0.15	6.07	±	0.01	100	6.10	±	0.00	0.0	±	0.0	6.13	±	0.01	0.0	±	0.0
MMI1_1129	16.84	±	0.74	5.75	±	0.00	100	6.20	±	0.01	100.0	±	0.0	6.32	±	0.00	53.6	±	8.0
MMI2_1130	13.01	±	0.11	5.84	±	0.00	100	6.12	±	0.00	39.7	±	2.3	6.28	±	0.00	15.3	±	4.0
MMS1_1143	22.11	±	1.38	5.70	±	0.01	100	6.21	±	0.00	30.7	±	8.0	6.74	±	0.00	27.7	±	2.3
*Debaryomyces* genus																			
25	8.27	±	0.27	6.15	±	0.04	100	6.06	±	0.02	59.5	±	2.3	5.86	±	0.03	0.0	±	0.0
AN4_1195	10.49	±	0.66	5.91	±	0.00	100	6.06	±	0.01	30.2	±	1.1	6.57	±	0.01	0.0	±	0.0
BAT1_1192	8.52	±	0.38	5.88	±	0.00	100	6.15	±	0.00	30.2	±	16.2	6.52	±	0.00	13.7	±	2.0
BAT2_1170	4.46	±	0.27	5.70	±	0.01	100	6.17	±	0.01	93.8	±	8.8	6.28	±	0.01	0.0	±	0.0
BAT2_1171	7.53	±	0.11	6.04	±	0.01	100	6.38	±	0.00	19.1	±	2.8	6.69	±	0.00	8.6	±	1.3
BAT2_1172	10.92	±	0.82	5.75	±	0.00	100	6.37	±	0.00	43.5	±	6.9	6.58	±	0.00	22.9	±	14.7
BB1_1197	11.94	±	0.03	5.41	±	0.01	100	6.11	±	0.00	0.0	±	0.0	6.62	±	0.00	0.0	±	0.0
BB2_1199	13.47	±	0.41	5.80	±	0.00	100	6.02	±	0.03	54.8	±	2.1	6.08	±	0.01	50.0	±	0.0
BB4_1204	15.17	±	0.24	5.49	±	0.00	100	5.91	±	0.00	100.0	±	0.0	6.38	±	0.00	0.0	±	0.0
BEM1_1173	13.34	±	1.81	5.88	±	0.01	100	6.17	±	0.00	49.0	±	25.0	6.32	±	0.00	21.7	±	10.5
BEM1_1174	12.78	±	0.03	6.16	±	0.00	100	6.10	±	0.01	74.5	±	5.1	6.30	±	0.00	4.5	±	0.7
BEM1_1175	14.13	±	0.10	5.10	±	0.01	100	6.32	±	0.00	53.4	±	1.8	6.46	±	0.00	0.0	±	0.0
BES1_1198	12.28	±	0.39	6.14	±	0.01	100	6.03	±	0.04	47.6	±	12.3	6.72	±	0.00	23.8	±	1.7
LAI2_1200	17.49	±	0.06	5.83	±	0.02	100	6.06	±	0.01	61.3	±	1.4	6.52	±	0.00	16.2	±	2.8
LAI3_1201	11.28	±	0.86	6.09	±	0.00	100	5.91	±	0.00	69.5	±	4.0	6.47	±	0.00	10.3	±	7.2
LAI3_1202	5.49	±	0.56	5.64	±	0.00	100	6.25	±	0.01	43.7	±	1.1	6.33	±	0.00	25.0	±	0.0
LAIF1_1167	13.20	±	0.56	5.97	±	0.00	100	6.34	±	0.00	89.3	±	15.2	6.66	±	0.00	33.0	±	3.0
LAIF1_1168	12.75	±	0.33	6.16	±	0.01	100	6.06	±	0.01	13.0	±	3.1	6.61	±	0.00	0.0	±	0.0
LAIF2_1169	14.05	±	0.69	6.23	±	0.01	100	6.12	±	0.01	19.3	±	2.9	6.56	±	0.00	0.0	±	0.0
MM1_1193	13.71	±	0.21	5.87	±	0.00	100	6.08	±	0.00	95.3	±	1.5	6.53	±	0.01	11.9	±	6.7
MM2_1177	4.35	±	0.53	5.80	±	0.00	100	5.97	±	0.00	8.8	±	1.6	6.12	±	0.01	0.0	±	0.0
MM2_1178	9.04	±	0.10	6.24	±	0.01	100	6.28	±	0.00	16.0	±	0.9	6.45	±	0.00	3.2	±	1.3
MM2_1179	14.10	±	0.04	5.94	±	0.00	100	6.14	±	0.00	4.5	±	0.0	6.39	±	0.00	0.0	±	0.0
MM3_1180	14.27	±	0.26	6.20	±	0.01	100	6.32	±	0.01	55.6	±	0.6	6.49	±	0.00	19.7	±	3.8
MM3_1181	12.38	±	0.06	5.79	±	0.00	100	6.18	±	0.01	47.4	±	3.7	6.32	±	0.02	0.0	±	0.0
MM3_1182	11.91	±	0.11	6.25	±	0.00	100	6.14	±	0.00	60.6	±	2.1	6.60	±	0.00	32.8	±	2.3
MM4_1184	7.96	±	1.17	5.97	±	0.00	100	6.21	±	0.01	57.6	±	2.2	6.62	±	0.01	27.8	±	7.9
MM6_1186	12.38	±	0.54	6.09	±	0.01	100	6.25	±	0.01	23.4	±	1.7	6.60	±	0.00	13.6	±	1.0
MM6_1187	6.76	±	1.08	5.58	±	0.00	100	6.38	±	0.00	58.6	±	4.7	6.38	±	0.00	18.3	±	2.4
MM6_1188	5.93	±	0.09	5.88	±	0.00	100	6.36	±	0.00	75.4	±	0.5	6.35	±	0.01	21.9	±	4.4
MM6_1194	15.63	±	0.09	6.09	±	0.04	100	6.06	±	0.00	0.0	±	0.0	6.09	±	0.00	0.0	±	0.0
MM7_1190	13.26	±	0.11	6.20	±	0.01	100	6.28	±	0.01	24.4	±	1.9	6.50	±	0.00	18.1	±	0.6
MM7_1191	11.04	±	0.28	6.35	±	0.00	100	6.29	±	0.00	44.2	±	1.0	6.16	±	0.00	19.3	±	9.2
MMF1_1176	4.20	±	0.23	6.21	±	0.01	100	6.36	±	0.01	60.2	±	1.9	6.33	±	0.00	15.5	±	2.3
MMS1_1196	19.36	±	0.02	6.19	±	0.00	100	6.31	±	0.01	93.7	±	3.4	6.46	±	0.00	56.4	±	6.2
PC1_1159	34.47	±	0.63	6.06	±	0.01	100	6.06	±	0.01	57.4	±	10.4	6.55	±	0.00	34.4	±	1.5
PC2_1160	1.13	±	0.20	5.80	±	0.00	100	6.36	±	0.00	31.7	±	5.1	6.50	±	0.00	20.6	±	13.4
PC2_1161	3.36	±	0.02	6.13	±	0.02	100	6.07	±	0.01	22.1	±	1.4	6.23	±	0.00	12.0	±	1.3
PC3_1163	12.49	±	0.38	5.75	±	0.00	100	6.05	±	0.00	37.3	±	2.2	6.27	±	0.00	0.0	±	0.0
PC5_1164	12.61	±	0.21	5.80	±	0.00	100	6.30	±	0.00	50.6	±	13.3	6.36	±	0.00	44.8	±	2.2
PC5_1165	14.34	±	0.44	6.36	±	0.00	100	6.40	±	0.00	11.7	±	1.2	6.67	±	0.00	5.8	±	2.5
PC5_1166	16.91	±	0.76	6.19	±	0.01	100	6.05	±	0.00	55.8	±	8.2	6.45	±	0.00	48.7	±	3.4
PCF1_1147	10.77	±	0.43	6.30	±	0.00	100	6.14	±	0.01	25.1	±	16.0	6.49	±	0.00	20.5	±	3.0
PCF1_1148	1.16	±	0.16	6.20	±	0.01	100	6.19	±	0.01	0.0	±	0.0	6.20	±	0.00	0.0	±	0.0
PCF1_1149	9.92	±	0.81	6.25	±	0.00	100	6.28	±	0.00	30.6	±	1.4	6.55	±	0.00	12.1	±	1.8
PCF2_1150	13.23	±	0.53	5.91	±	0.00	100	6.27	±	0.00	40.7	±	3.1	6.75	±	0.00	6.2	±	2.2
PCF2_1151	1.33	±	0.21	5.87	±	0.00	100	5.84	±	0.00	18.8	±	8.8	6.37	±	0.00	0.0	±	0.0
PCI1_1156	7.71	±	0.19	6.03	±	0.00	100	6.40	±	0.00	60.0	±	0.0	6.50	±	0.00	7.4	±	0.8
PCI1_1157	7.92	±	0.08	6.29	±	0.00	100	6.28	±	0.00	64.0	±	25.2	6.51	±	0.00	55.1	±	1.0
PCI2_1158	17.64	±	0.80	5.91	±	0.00	100	6.20	±	0.00	13.4	±	1.3	6.53	±	0.00	0.0	±	0.0
PCS1_1152	11.10	±	0.77	5.94	±	0.00	100	6.17	±	0.00	73.2	±	1.8	6.24	±	0.00	55.2	±	19.8
PCS1_1153	9.29	±	0.49	6.21	±	0.01	100	6.50	±	0.01	9.2	±	5.8	6.40	±	0.00	6.9	±	3.8
PCS2_1154	14.39	±	0.26	6.32	±	0.00	100	6.14	±	0.01	23.3	±	0.3	6.58	±	0.00	12.7	±	2.2
PCS2_1155	2.38	±	0.43	5.70	±	0.00	100	6.14	±	0.00	86.3	±	31.1	6.74	±	0.00	82.1	±	1.8
*Hanseniaspora* genus																			
Mb	39.72	±	0.84	5.94	±	0.04	100	5.97	±	0.04	0.0	±	0.0	5.92	±	0.07	0.0	±	0.0
LV8	35.84	±	1.10	6.10	±	0.03	100	5.60	±	0.14	41.5	±	1.1	6.22	±	0.01	0.0	±	0.0
LV12	42.36	±	1.67	6.13	±	0.08	100	6.10	±	0.11	15.1	±	3.6	6.10	±	0.03	0.0	±	0.0
*Kazachstania* genus																			
m3-4	51.07	±	1.16	5.18	±	0.12	100	5.34	±	0.09	58.3	±	11.8	5.91	±	0.05	0.0	±	0.0
m3-5	48.44	±	2.01	5.61	±	0.05	100	5.96	±	0.02	0.0	±	0.0	6.05	±	0.03	0.0	±	0.0
m3-6	48.50	±	1.44	5.95	±	0.06	100	5.96	±	0.02	13.8	±	0.7	6.52	±	0.01	0.0	±	0.0
m3-7	52.51	±	1.30	6.00	±	0.04	100	5.89	±	0.02	48.7	±	1.9	6.06	±	0.02	0.0	±	0.0
m3-A3	62.28	±	0.34	6.18	±	0.01	100	6.14	±	0.00	57.2	±	2.3	6.04	±	0.02	22.7	±	0.0
m3-B3	62.34	±	0.58	6.22	±	0.01	100	6.17	±	0.01	89.4	±	2.7	6.22	±	0.01	56.6	±	1.5
m3-C3	53.14	±	0.27	5.82	±	0.03	100	5.61	±	0.05	31.0	±	3.4	5.72	±	0.04	11.8	±	1.0
*Kluyveromyces* genus																			
101	36.61	±	0.44	6.15	±	0.07	100	6.15	±	0.11	0.0	±	0.0	6.08	±	0.05	0.0	±	0.0
102	44.88	±	0.32	6.12	±	0.06	100	6.11	±	0.09	0.0	±	0.0	6.08	±	0.11	0.0	±	0.0
103	55.57	±	0.77	6.04	±	0.05	100	6.07	±	0.06	26.6	±	4.0	6.10	±	0.11	10.0	±	2.4
104	30.25	±	0.62	6.07	±	0.03	100	6.01	±	0.02	38.0	±	0.7	6.39	±	0.01	12.1	±	0.5
105	64.19	±	0.42	5.99	±	0.02	100	5.89	±	0.02	38.8	±	1.8	5.99	±	0.02	24.0	±	1.4
106	14.58	±	0.27	5.79	±	0.06	100	5.67	±	0.04	40.2	±	3.8	5.72	±	0.04	35.4	±	2.9
Md	60.92	±	0.82	6.01	±	0.06	100	5.96	±	0.02	0.0	±	0.0	6.03	±	0.04	0.0	±	0.0
Mf	66.37	±	0.55	6.07	±	0.06	100	6.07	±	0.03	0.0	±	0.0	6.05	±	0.07	0.0	±	0.0
*Lachancea* genus																			
B8	70.06	±	0.88	5.61	±	0.05	100	5.72	±	0.04	35.6	±	0.7	6.36	±	0.01	0.0	±	0.0
B13	70.89	±	0.79	5.77	±	0.03	100	5.86	±	0.03	35.6	±	1.1	6.15	±	0.01	0.0	±	0.0
B15	32.12	±	0.19	6.04	±	0.02	100	6.09	±	0.02	35.9	±	1.3	6.28	±	0.01	26.2	±	0.6
B57	38.61	±	0.73	6.09	±	0.17	100	6.07	±	0.14	0.0	±	0.0	6.06	±	0.13	0.0	±	0.0
*Metschnikowia* genus																			
B27	66.21	±	2.05	5.92	±	0.07	100	5.77	±	0.03	52.8	±	3.9	6.17	±	0.01	4.3	±	0.1
B28	43.60	±	1.63	6.04	±	0.05	100	6.01	±	0.02	0.0	±	0.0	6.04	±	0.05	0.0	±	0.0
B33	60.12	±	0.94	6.13	±	0.11	100	6.11	±	0.05	0.0	±	0.0	6.11	±	0.01	0.0	±	0.0
B42	11.65	±	0.28	6.06	±	0.05	100	6.08	±	0.08	0.0	±	0.0	6.03	±	0.04	0.0	±	0.0
B49	54.07	±	1.34	6.10	±	0.14	100	6.08	±	0.05	0.0	±	0.0	6.10	±	0.03	0.0	±	0.0
F14	28.61	±	0.89	6.00	±	0.08	100	6.01	±	0.02	0.0	±	0.0	6.07	±	0.06	0.0	±	0.0
G3	29.34	±	0.43	5.93	±	0.02	100	5.53	±	0.06	47.2	±	3.9	5.72	±	0.04	55.0	±	7.1
G6	55.89	±	0.72	6.01	±	0.06	100	6.03	±	0.23	0.0	±	0.0	5.96	±	0.17	0.0	±	0.0
*Pichia* genus							100												
18	20.31	±	0.32	5.89	±	0.02	100	5.45	±	0.07	24.4	±	0.8	6.11	±	0.01	0.0	±	0.0
E1	74.26	±	1.44	6.10	±	0.08	100	6.10	±	0.03	0.0	±	0.0	6.05	±	0.07	0.0	±	0.0
Ma	77.02	±	1.10	6.03	±	0.09	100	6.02	±	0.07	0.0	±	0.0	6.06	±	0.08	0.0	±	0.0
Mg	72.41	±	0.78	6.03	±	0.04	100	6.07	±	0.06	0.0	±	0.0	6.04	±	0.02	0.0	±	0.0
*Rhodotorula* genus							100												
B5	19.08	±	0.73	5.89	±	0.02	100	5.77	±	0.03	10.6	±	0.8	5.77	±	0.03	0.0	±	0.0
B14	56.81	±	0.94	5.34	±	0.09	100	5.67	±	0.04	13.4	±	1.3	5.89	±	0.02	0.0	±	0.0
F5	34.94	±	2.51	6.10	±	0.11	100	6.08	±	0.05	0.0	±	0.0	6.06	±	0.02	0.0	±	0.0
*Saccharomyces* genus																			
46	39.62	±	0.69	6.17	±	0.04	100	6.17	±	0.01	14.3	±	0.0	5.94	±	0.00	0.0	±	0.0
B6	63.16	±	1.80	6.15	±	0.01	100	6.20	±	0.04	39.4	±	3.3	6.19	±	0.05	20.1	±	2.3
m1-1	58.23	±	0.35	6.06	±	0.02	100	6.09	±	0.02	69.8	±	2.3	6.13	±	0.01	15.4	±	0.6
m1-2	54.44	±	1.68	6.09	±	0.02	100	6.11	±	0.01	63.9	±	1.9	6.17	±	0.01	19.5	±	0.7
m1-3	61.83	±	1.04	6.03	±	0.04	100	6.04	±	0.02	28.6	±	1.2	6.24	±	0.01	0.0	±	0.0
m1-4	46.12	±	1.61	6.05	±	0.03	100	6.11	±	0.01	19.5	±	0.7	6.36	±	0.01	11.0	±	0.2
m1-5	55.47	±	0.72	5.99	±	0.02	100	6.01	±	0.02	64.6	±	2.9	5.99	±	0.02	0.0	±	0.0
m1-6	55.18	±	0.13	5.72	±	0.04	100	6.01	±	0.02	60.7	±	2.6	6.03	±	0.04	23.6	±	2.0
m1-7	62.05	±	0.99	5.97	±	0.04	100	5.99	±	0.02	45.2	±	2.1	6.43	±	0.01	13.8	±	0.2
m1-8	57.45	±	2.08	5.86	±	0.03	100	6.28	±	0.01	8.7	±	0.5	6.25	±	0.01	0.0	±	0.0
m1-9	53.45	±	2.06	6.06	±	0.02	100	6.09	±	0.02	78.9	±	3.4	6.01	±	0.02	10.3	±	0.4
m2-1	57.97	±	1.03	5.45	±	0.07	100	5.67	±	0.04	45.0	±	7.1	5.86	±	0.03	40.2	±	3.8
m2-2	54.28	±	0.13	5.93	±	0.02	100	6.28	±	0.01	51.9	±	2.7	6.24	±	0.01	49.2	±	1.1
m2-3	65.41	±	0.69	5.99	±	0.02	100	6.01	±	0.02	36.4	±	1.6	6.06	±	0.02	27.0	±	1.0
7V	65.58	±	0.80	6.22	±	0.01	100	6.28	±	0.01	82.0	±	1.9	6.45	±	0.01	4.4	±	0.1
8C	64.71	±	0.73	6.20	±	0.01	100	6.45	±	0.00	60.0	±	0.0	6.54	±	0.01	54.1	±	0.7
10C	66.49	±	0.41	6.24	±	0.01	100	6.40	±	0.01	69.4	±	0.8	6.58	±	0.01	27.2	±	0.5
8	66.40	±	0.78	6.23	±	0.03	100	6.18	±	0.01	46.9	±	0.8	6.40	±	0.01	20.4	±	0.6
2	47.33	±	0.10	5.95	±	0.06	100	6.00	±	0.04	62.7	±	5.5	6.43	±	0.01	34.5	±	0.6
5V	56.08	±	0.13	6.09	±	0.02	100	6.25	±	0.01	56.4	±	2.0	6.22	±	0.01	45.6	±	1.1
6	66.40	±	0.71	6.21	±	0.02	100	6.38	±	0.01	37.3	±	0.9	6.27	±	0.01	28.6	±	0.5
4PV	59.40	±	1.63	6.15	±	0.01	100	6.41	±	0.01	91.6	±	1.6	6.49	±	0.01	90.9	±	1.3
2PV	62.81	±	1.94	6.10	±	0.03	100	6.31	±	0.01	49.2	±	1.1	6.38	±	0.01	18.2	±	0.3
5	58.55	±	1.69	6.02	±	0.07	100	6.01	±	0.02	43.3	±	1.7	6.06	±	0.02	0.0	±	0.0
1PV	64.42	±	0.65	6.20	±	0.01	100	6.47	±	0.01	80.4	±	1.1	6.52	±	0.01	29.5	±	0.4
7	66.41	±	1.50	5.99	±	0.02	100	6.12	±	0.06	44.1	±	0.5	6.60	±	0.00	19.4	±	0.9
4	60.69	±	0.79	6.13	±	0.01	100	6.18	±	0.01	27.9	±	0.9	6.13	±	0.01	12.3	±	0.4
CODEX	65.58	±	2.83	6.17	±	0.01	100	6.17	±	0.04	42.7	±	3.9	6.22	±	0.03	22.7	±	1.8
*Torulaspora* genus																			
1C	40.08	±	1.60	6.12	±	0.03	100	6.20	±	0.00	52.8	±	1.4	6.45	±	0.01	13.3	±	0.4
1E	36.65	±	0.09	5.94	±	0.04	100	6.10	±	0.00	59.5	±	2.3	6.25	±	0.01	10.5	±	0.3
2A	68.90	±	1.53	5.86	±	0.03	100	6.00	±	0.00	69.7	±	4.3	6.32	±	0.01	26.9	±	0.6
3H	59.14	±	1.25	5.97	±	0.08	100	6.40	±	0.00	8.2	±	0.2	6.40	±	0.01	0.0	±	0.0
4E	63.61	±	5.71	6.17	±	0.04	100	6.30	±	0.00	47.5	±	1.1	6.39	±	0.01	10.1	±	0.2
5D	76.13	±	0.18	5.86	±	0.03	100	5.90	±	0.00	10.3	±	0.4	6.09	±	0.02	0.0	±	0.0
B7	69.21	±	0.94	6.06	±	0.02	100	6.10	±	0.00	35.8	±	0.8	6.32	±	0.01	4.9	±	0.2
1.1t2	66.76	±	0.74	5.91	±	0.05	100	6.00	±	0.00	45.2	±	2.1	6.25	±	0.01	35.1	±	0.9
2.2t1	57.07	±	1.01	6.15	±	0.01	100	6.20	±	0.00	61.3	±	1.8	6.13	±	0.01	9.3	±	0.3
7.3t2	72.97	±	0.77	6.00	±	0.04	100	6.10	±	0.00	61.6	±	2.2	5.99	±	0.02	19.4	±	0.9
7.3t0	61.26	±	0.23	5.89	±	0.02	100	5.90	±	0.00	74.2	±	3.9	6.13	±	0.01	23.3	±	0.8
12.2t2	55.84	±	1.01	6.01	±	0.02	100	6.10	±	0.00	83.0	±	2.9	6.43	±	0.01	39.1	±	0.6
15.2t2	42.99	±	1.74	6.10	±	0.03	100	6.20	±	0.00	77.0	±	0.8	6.63	±	0.00	45.9	±	2.7
19.1t2	35.04	±	0.06	5.91	±	0.05	100	6.00	±	0.00	18.2	±	0.2	6.58	±	0.01	6.5	±	0.3
19.2t2	67.11	±	1.24	6.04	±	0.02	100	6.10	±	0.00	47.3	±	1.2	6.24	±	0.01	25.7	±	0.9
19.3t2	63.40	±	0.29	5.96	±	0.02	100	6.00	±	0.10	23.0	±	0.4	6.43	±	0.01	6.9	±	0.3
19.4t0	25.83	±	0.20	6.17	±	0.05	100	6.30	±	0.00	56.7	±	1.2	6.36	±	0.01	43.8	±	0.8
33	54.93	±	1.30	6.21	±	0.07	100	6.20	±	0.00	0.0	±	0.0	6.18	±	0.03	0.0	±	0.0
34	52.29	±	1.05	6.17	±	0.04	100	6.30	±	0.00	57.6	±	1.4	6.59	±	0.01	16.1	±	0.4
35	67.41	±	1.59	6.20	±	0.01	100	6.60	±	0.00	73.6	±	0.8	6.70	±	0.00	7.5	±	0.1
37	58.90	±	0.18	5.86	±	0.03	100	5.70	±	0.00	45.9	±	1.1	6.28	±	0.01	0.0	±	0.0
38	47.23	±	0.31	5.45	±	0.07	100	5.60	±	0.00	0.0	±	0.0	5.86	±	0.03	0.0	±	0.0
39	67.24	±	1.37	5.91	±	0.05	100	5.90	±	0.00	51.2	±	1.7	6.13	±	0.01	35.9	±	3.6
40	62.66	±	0.94	6.19	±	0.05	100	6.20	±	0.00	34.1	±	1.0	6.17	±	0.01	29.7	±	1.6
92	29.35	±	0.34	5.89	±	0.02	100	5.80	±	0.00	47.7	±	3.2	6.13	±	0.01	18.6	±	0.6
94	51.55	±	0.65	6.14	±	0.06	100	6.20	±	0.00	28.6	±	0.8	6.17	±	0.01	25.5	±	0.8
6809	57.07	±	1.31	6.02	±	0.07	100	6.20	±	0.00	17.0	±	0.5	6.44	±	0.01	9.0	±	0.1
C 7.4	71.92	±	0.83	6.20	±	0.04	100	6.30	±	0.00	52.3	±	1.1	6.45	±	0.01	19.8	±	0.3
J401	73.51	±	1.34	5.86	±	0.03	100	5.90	±	0.00	48.1	±	2.7	6.18	±	0.01	0.0	±	0.0
Td vcs ff	71.54	±	1.53	6.10	±	0.03	100	6.20	±	0.00	56.1	±	3.2	6.52	±	0.01	11.2	±	0.1

DPPH, 1,1-Diphenyl-2-Picrylhydrazyl.

**Table 4:** Antimicrobial activity of the yeast strains isolated from NE and SPF. Five human pathogenic bacteria were chosen for the test. The “+”indicates the ability of the yeast to inhibit bacterial growth in a double-layer agar test, the “±” indicates faint inhibition, and the “−“ indicates no inhibition action by yeast.

Yeast Strains	Human Pathogenic Bacteria				
	*C. albicans*	*E. coli*	*L. monocytogenes*	*S. aureus*	*S. enterica*
*Brettanomyces* genus					
G2	+	+	−	±	±
G4	+	+	−	±	±
G6	+	+	±	+	+
G8	+	+	−	+	+
*Candida* genus					
7	+	+	±	+	+
28	+	+	±	+	+
AN4_1127	−	−	−	−	−
B9	+	+	±	+	+
B10	+	+	±	+	+
B29	+	+	±	+	+
BAT1_1144	−	−	±	−	−
BB2_1145	−	−	−	−	±
LAIF1_1123	−	−	+	−	−
LAIF1_1124	−	−	−	−	−
LAIF1_1125	−	−	−	−	−
LAIF1_1126	−	−	−	−	−
MM1_1132	−	−	−	−	−
MM2_1133	−	−	−	−	±
MM2_1140	±	±	−	−	±
MM2_1141	−	−	−	−	−
MM4_1135	−	−	−	−	−
MM4_1136	−	±	±	−	−
MMF1_1128	−	−	±	−	±
MMF1_1142	+	±	±	−	−
MMF2_1137	−	−	±	−	±
MMF2_1138	−	−	−	−	±
MMI1_1129	−	−	−	−	−
MMI2_1130	−	−	−	−	±
MMS1_1143	−	±	±	−	−
*Debaryomyces* genus					
25	+	+	+	+	+
AN4_1195	−	−	−	−	−
BAT1_1192	−	−	−	−	−
BAT2_1170	−	−	−	−	−
BAT2_1171	−	−	−	−	−
BAT2_1172	−	+	+	+	+
BB1_1197	−	±	−	−	±
BB2_1199	−	+	+	+	+
BB4_1204	−	−	−	−	±
BEM1_1173	−	−	−	−	−
BEM1_1174	−	±	−	−	±
BEM1_1175	−	+	+	+	+
BES1_1198	±	+	±	−	±
LAI2_1200	−	−	−	−	−
LAI3_1201	−	−	−	−	−
LAI3_1202	−	−	−	−	−
LAIF1_1167	−	−	−	−	±
LAIF1_1168	−	−	−	−	±
LAIF2_1169	−	−	−	−	−
MM1_1193	−	−	−	−	−
MM2_1177	−	−	−	−	±
MM2_1178	−	−	−	−	−
MM2_1179	−	−	−	−	−
MM3_1180	−	−	−	−	−
MM3_1181	−	±	−	−	±
MM3_1182	−	−	−	−	−
MM4_1184	−	−	−	−	−
MM6_1186	−	−	−	−	−
MM6_1187	−	−	−	−	−
MM6_1188	−	−	−	−	±
MM6_1194	−	−	−	−	−
MM7_1190	−	−	−	−	−
MM7_1191	−	−	−	−	−
MMF1_1176	−	−	−	−	−
MMS1_1196	−	±	−	−	−
PC1_1159	−	−	−	−	−
PC2_1160	+	±	−	−	±
PC2_1161	−	−	−	−	−
PC3_1163	−	−	−	−	−
PC5_1164	−	−	−	−	−
PC5_1165	−	+	±	−	+
PC5_1166	−	−	±	−	±
PCF1_1147	−	−	−	−	−
PCF1_1148	−	−	−	−	−
PCF1_1149	−	−	−	−	−
PCF2_1150	−	−	−	−	−
PCF2_1151	−	−	−	−	−
PCI1_1156	−	−	−	−	−
PCI1_1157	−	−	−	−	±
PCI2_1158	−	−	−	−	±
PCS1_1152	−	−	−	−	−
PCS1_1153	−	−	−	−	−
PCS2_1154	±	−	−	−	−
PCS2_1155	−	−	−	−	−
*Hanseniaspora* genus					
Mb	+	−	−	±	−
LV8	+	+	−	+	−
LV12	+	+	−	+	−
*Kazachstania* genus					
m3-4	+	+	±	+	+
m3-5	+	+	±	+	+
m3-6	+	+	−	+	+
m3-7	+	+	−	+	+
m3-A3	+	+	−	+	+
m3-B3	+	+	±	+	+
m3-C3	+	+	−	+	+
*Kluyveromyces* genus					
101	+	+	±	+	+
102	+	+	±	+	+
103	+	+	−	+	+
104	+	+	±	+	+
105	+	+	−	+	+
106	+	+	±	+	+
Md	+	+	−	+	+
Mf	+	+	±	+	+
*Lachancea* genus					
B8	+	+	±	+	+
B13	+	+	−	+	+
B15	+	+	−	+	+
B57	+	+	−	+	+
*Metschnikowia* genus					
B27	+	+	±	+	+
B28	+	+	−	+	±
B33	+	+	±	+	+
B42	+	±	±	+	+
B49	+	+	−	+	+
F14	+	+	−	+	+
G3	+	±	−	±	−
G6	±	±	−	−	−
*Pichia* genus					
18	±	+	±	+	+
E1	+	+	−	+	+
Ma	±	+	±	±	±
Mg	±	+	±	±	±
*Rhodotorula* genus					
B5	+	−	−	±	−
B14	−	−	−	±	−
F5	−	−	−	−	−
*Saccharomyces* genus					
46	+	+	−	+	+
B6	+	+	−	+	+
m1-1	+	+	±	+	+
m1-2	+	+	±	+	+
m1-3	+	+	±	+	+
m1-4	+	+	−	+	+
m1-5	+	+	±	+	+
m1-6	+	+	−	+	+
m1-7	+	+	−	+	+
m1-8	+	+	−	+	+
m1-9	+	+	−	+	+
m2-1	+	+	−	+	+
m2-2	+	+	−	+	+
m2-3	+	+	±	+	+
7V	+	+	±	+	+
8C	+	+	±	+	+
10C	+	+	−	+	+
8	+	+	+	+	+
2	+	+	+	+	+
5V	+	+	±	+	+
6	+	+	+	+	+
4PV	+	+	+	+	+
2PV	+	+	±	+	+
5	+	+	+	+	+
1PV	+	+	±	+	+
7	+	+	±	+	+
4	+	+	±	+	+
CODEX	+	+	−	+	+
*Torulaspora* genus					
1C	+	+	±	+	+
1E	+	+	−	+	+
2A	+	+	±	+	+
3H	+	+	±	+	+
4E	+	+	±	+	+
5D	+	+	−	+	+
B7	+	+	±	+	+
1.1t2	+	+	−	+	+
2.2t1	+	+	−	+	+
7.3t2	+	+	±	+	+
7.3t0	+	+	±	+	+
12.2t2	+	+	−	+	+
15.2t2	+	+	±	+	+
19.1t2	+	+	−	+	+
19.2t2	+	+	±	+	+
19.3t2	+	+	±	+	+
19.4t0	+	+	±	+	+
33	+	+	−	+	+
34	+	+	±	+	+
35	+	+	±	+	+
37	+	+	±	+	+
38	+	−	−	−	−
39	+	+	±	+	+
40	+	+	−	+	+
92	+	+	−	+	+
94	+	+	−	+	+
6809	+	+	±	+	+
C 7.4	+	+	±	+	+
J401	+	+	−	+	+
Td vcs ff	+	+	±	+	+
